# Dietary Inflammatory Index and Incidence of Cardiovascular Disease in the PREDIMED Study

**DOI:** 10.3390/nu7064124

**Published:** 2015-05-29

**Authors:** Ana Garcia-Arellano, Raul Ramallal, Miguel Ruiz-Canela, Jordi Salas-Salvadó, Dolores Corella, Nitin Shivappa, Helmut Schröder, James R. Hébert, Emilio Ros, Enrique Gómez-Garcia, Ramon Estruch, José Lapetra, Fernando Arós, Miquel Fiol, Lluis Serra-Majem, Xavier Pintó, Nancy Babio, José I. González, Montse Fitó, J. Alfredo Martínez, Miguel A. Martínez-González, the PREDIMED investigators

**Affiliations:** 1 Department of Emergency, Complejo Hospitalario de Navarra, Servicio Navarro de Salud, Pamplona 31008, Spain; E-Mail: agarare@gmail.com; 2 The PREDIMED (Prevención con Dieta Mediterránea) Research Network (RD 06/0045), Instituto de Salud Carlos III, Madrid 28029, Spain; E-Mails: mcanela@unav.es (M.R.-C.); jordi.salas@urv.cat (J.S.-S.); egomezgracia@uma.es (E.G.-G.); restruch@clinic.ub.es (R.E.); aborau@secardiologia.es (F.A.); lserra@dcc.ulpgc.es (L.S.-M.); xpinto@bellvitgehospital.cat (X.P.); nancy.babio@urv.cat (N.B.); mamartinez@unav.es (M.M.-G.); 3 Centro de Investigación Biomédica en Epidemiología y Salud Pública, Madrid 28029, Spain; E-Mails: dolores.corella@uv.es (D.C.); HSchoeder@imim.es (H.S.); EROS@clinic.ub.es (E.R.); jlapetra@ono.com (J.L.); miquelfiol@yahoo.es (M.F.); arraez@uv.es (J.I.G.); MFITO@imim.es (M.F.); 4 Department of Preventive Medicine and Public Health, School of Medicine, University of Navarra, Pamplona 31008, Spain; E-Mail: raulramallal@hotmail.com; 5 Department of Cardiology, Complejo Hospitalario de Navarra, Servicio Navarro de Salud, Pamplona 31008, Spain; 6 Human Nutrition Department, Hospital Universitari Sant Joan, Institut d’Investigació Sanitaria Pere Virgili, Universitat Rovira i Virgili, Reus 43201, Spain; 7 Department of Preventive Medicine, University of Valencia, Valencia 46071, Spain; 8 South Carolina Statewide Cancer Prevention and Control Program, University of South Carolina, Columbia, SC, 29208 USA; E-Mails: shivappa@email.sc.edu (N.S.); JHEBERT@mailbox.sc.edu (J.H.); 9 Cardiovascular and Nutrition Research Group, Institut de Recerca Hospital del Mar, Barcelona 08003, Spain; 10 Lipid Clinic, Department of Endocrinology and Nutrition, Institut d’Investigacions Biomèdiques August Pi i Sunyer, Hospital Clinic, University of Barcelona, Barcelona 08036, Spain; 11 Department of Preventive Medicine, University of Malaga, Malaga 29010, Spain; 12 Department of Internal Medicine Institut d’Investigacions Biomèdiques August Pi i Sunyer, Hospital Clinic, University of Barcelona, Barcelona 08036, Spain; 13 Department of Family Medicine, Primary Care Division of Seville, San Pablo Health Center, Seville 41007, Spain; 14 Department of Cardiology, University Hospital of Alava, Vitoria 01009, Spain; 15 Institute of Health Sciences (IUNICS), University of Balearic Islands, and Hospital Son Espases, Palma de Mallorca 07120, Spain; 16 Research Institut of Biomedical and Health Sciences, University of Las Palmas de Gran Canaria, Las Palmas 35001, Spain; 17 Lipids and Vascular Risk Unit, Internal Medicine, Hospital Universitario de Bellvitge, Hospitalet de Llobregat, Barcelona 08907, Spain; 18 Department of Physiology and Nutrition, University of Navarra, Pamplona 31008, Spain

**Keywords:** dietary inflammatory index, cardiovascular disease, PREDIMED, inflammation

## Abstract

Previous studies have reported an association between a more pro-inflammatory diet profile and various chronic metabolic diseases. The Dietary Inflammatory Index (DII) was used to assess the inflammatory potential of nutrients and foods in the context of a dietary pattern. We prospectively examined the association between the DII and the incidence of cardiovascular disease (CVD: myocardial infarction, stroke or cardiovascular death) in the PREDIMED (Prevención con Dieta Mediterránea) study including 7216 high-risk participants. The DII was computed based on a validated 137-item food frequency questionnaire. Multivariate-adjusted hazard ratios (HR) and 95% confidence intervals of CVD risk were computed across  quartiles of the DII where the lowest (most anti-inflammatory) quartile is the referent. Risk increased across the quartiles (*i.e.*, with increasing inflammatory potential): HR_quartile2_ = 1.42 (95%CI = 0.97–2.09); HR_quartile3_ = 1.85 (1.27–2.71); and HR_quartile4_ = 1.73 (1.15–2.60). When fit as continuous the multiple-adjusted hazard ratio for each additional standard deviation of the DII was 1.22 (1.06–1.40). Our results provide direct prospective evidence that a pro-inflammatory diet is associated with a higher risk of cardiovascular clinical events.

## 1. Introduction

Cardiovascular diseases (CVD) are the largest cause of morbidity and mortality in the world. It is expected that about 25 million people will die due from CVD, especially ischemic heart disease and stroke, in 2030 [1]. Atherosclerosis is the main cause of cardiovascular diseases, and inflammation is well known to be linked to the development and progression of atherosclerosis [2]. Inflammation is involved in all phases of the atherothrombotic process. Not only does promote the onset of the vascular injury, but it also leads to the progression and development of atherothrombotic complications that are responsible for acute ischemic clinical events [3].

The relationship between diet and CVDs is well recognized. Diet has been shown to modulate inflammation [4,5]. The Western dietary pattern, rich in red meat, refined grains, butter, processed meat, high-fat dairy products, sweets and desserts, potatoes, eggs, hydrogenated fats and sugared-sweetened beverages, has been associated with increased levels of inflammatory and other intermediate markers of CVD [4]. On the other hand, a Mediterranean dietary pattern (rich in olive oil, nuts, fruits and vegetables, whole grains and fish, moderate intake of alcohol, but low in red/processed meat, refined grains and sweets) has been reported to be associated with lower levels of inflammation and a lower risk of CVD [6,7,8,9,10].

Dietary patterns have been studied because they better describe the eating habits actually followed by a population and they take into account possible interactions between nutrients. Food patterns also may overcome potential confounding by specific nutrients or foods, and avoid the problem of collinearity between foods. However, the large variety of foods available in an average diet results in a large number of nutrients that can interact with each other, producing synergistic or antagonistic effects. Overall dietary scores allow a better assessment of the dietary pattern. In this context, the Dietary Inflammatory Index (DII) was proposed to assess the inflammatory effect of an individual’s diet [11]. The DII represents a literature-derived, population-based dietary score summarizing the effect of dietary parameters on six inflammatory biomarkers according to a comprehensive review of the published literature.

In addition to higher levels of inflammatory biomarkers, subjects consuming a pro-inflammatory diet, as represented by a higher DII, had increased indices of general and abdominal obesity, as previously reported [12]. The purpose of the present study was to prospectively examine the association between the DII values and the incidence of CVD during the follow-up interventions in the PREDIMED study.

## 2. Materials and Methods

The PREDIMED study (Prevención con Dieta Mediterránea) is a parallel group, multicenter, randomized trial conducted in Spain. The design, methods, and objectives of the PREDIMED study have been reported previously [13,14].

### 2.1. Participants

Briefly, participants were men (55 to 80 years of age) and women (60 to 80 years of age) with high cardiovascular risk, but with no history of clinical cardiovascular disease at enrollment. They were randomly assigned to one of three diets (a Mediterranean diet supplemented with extra-virgin olive oil, a Mediterranean diet supplemented with nuts, or a low-fat diet in the control group). Participants received individual and group educational sessions on a quarterly basis-, and, depending on the group assignment, free provision of extra-virgin olive oil, mixed nuts, or small nonfood gifts.

The protocol of the study was approved by the institutional review boards at all study locations.

The study began on 1 October 2003, and though the follow-up was planned to last for 6 years on average, on advice by the Data and Safety Monitoring Board, the trial was stopped early after a median follow-up of 4.8 years, on the basis of the results of the fourth interim analysis, which showed early evidence of benefit [15].

### 2.2. Data Collection and Outcomes

A 137-item validated food-frequency questionnaire was administered on a yearly basis. Biomarkers of compliance were measured in random subsamples of participants at one, three and five years and demonstrated adequate compliance with the intended diet in the intervention groups [15,16].

The primary end point was a composite of myocardial infarction, stroke, and death from cardiovascular causes. Secondary end-points were stroke, myocardial infarction, death from cardiovascular causes and death from any cause. Contacts with participants, contacts with family physicians, a yearly review of medical records, and consultation of the National Death Index were the sources used to identify primary and secondary end points. The medical records related to end points were examined by the end-point adjudication committee, whose members were unaware of the study-group assignments. Only end points that were confirmed by the adjudication committee and had occurred between 1 October 2003, and 1 December 2010, were included in the analysis.

The information derived from the 137-item Food-Frequency Questionnaire (FFQ) was used to calculate Dietary Inflammatory Index (DII) scores. The procedure used to calculate the DII scores for all subjects, from the FFQ, was described elsewhere [11]. Briefly, the DII was derived after a literature review from 1950 to 2010, including all articles that had assessed the role of whole foods and dietary constituents on interleukins (IL-1B, IL-4, IL-6, IL-10), Tumor Necrosis Factor-alpha and highly sensitive C-Reactive Protein (CRP). Overall DII scores for each participant represent the sum of each of the DII components in relation to the comparison global diet database. The DII score characterizes an individual’s diet on a continuum from maximally anti-inflammatory (negative values, lower quartiles) to maximally pro-inflammatory (positive values, higher quartiles) [17].

### 2.3. Statistical Analysis

**Figure 1:**
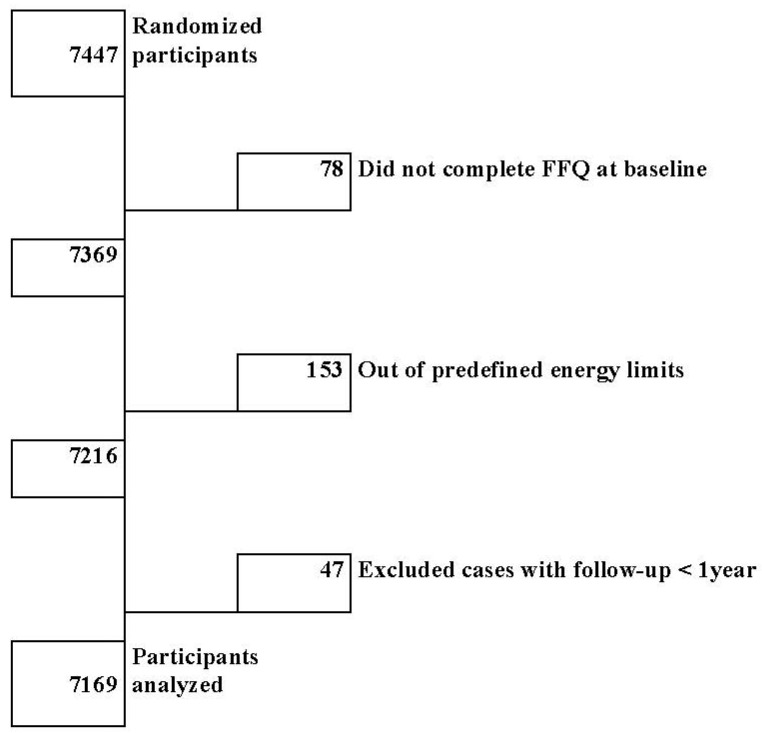
Flow chart of participant selection.

Participants were categorized into quartiles based on DII scores. Quartile sample size to achieve sufficient power was estimated assuming a comparison between extreme quartiles with two-tailed alpha error = 0.05, relative risk = 0.60, absolute risk (cumulative incidence) = 4% for two-quartile average (3% in the lowest *versus* 5% in the highest quartile), and statistical power = 0.80. Under these assumptions, the required sample size in each of the two extreme quartiles was 1605, which is covered with the number of participants in each of our extreme quartiles. Participants with total energy intake outside of predefined limits (800 and 4000 Kcal day^−1^ for men and 500 and 3500 Kcal day^−1^ for women) were excluded (Figure 1).

Time-to-event data were analyzed using Cox regression models. The time of the event was defined as the number of days from recruitment to the last visit, the diagnosis of the clinical cardiovascular event, or death (whichever came first) as determined by the external board of adjudicators of clinical events. Cox proportional hazard analyses were conducted with stratification for center and intervention group in all models. After a crude analysis, we fitted a model adjusted for sex and age. In a subsequent model we additionally adjusted for the major risk factors of cardiovascular disease. Robust standard errors were used. All p values were two-tailed. Statistical significance was set at the conventional 0.05 level.

## 3. Results

After exclusions, we included 7216 of the 7447 participants of the PREDIMED trial in our analysis (Figure 1). Among them, we observed 277 cardiovascular clinical events during 31,040 persons-years of follow-up.

Table 1 shows the main baseline characteristics of participants according to DII quartiles. The mean age of participants was 67.0 (SD: 6.2) years, and 57.4% were women. Participants in the higher quartiles of the DII had lower levels of leisure-time physical activity but also lower levels of dyslipidemia at baseline. The educational level was higher in participants whose values of the DII were lower, whereas their total energy intake was considerably higher. Carbohydrate intake, Polyunsaturated fatty acid (PUFA) intake, fiber intake and total intake of alcohol of participants in the lowest quartile of the DII were higher than in participants in the highest quartile.

**Table 1:** Description of main characteristics of participants according to the dietary inflammatory index score, the PREDIMED (Prevención con Dieta Mediterránea) trial, 2003–2010.

Variables	Quartiles of Dietary Inflammatory Index (DII) (a Higher Value Implies Higher Inflammatory Potential)			
	Q1	Q2	Q3	Q4
DII, median	−2.46	−1.34	−0.32	1.17
Number	1804	1804	1804	1804
Age, year	66 (6)	67 (6)	67 (6)	68 (6)
Sex (% Women),	55	57	58	60
Family history of early CHD, %	23	24	22	20
Hypertension, %	81	83	84	83
Dyslipidemia, %	76	72	71	69
Diabetes, %	48	46	49	52
Smoking, %				
Never	61	60	62	63
Former	26	26	24	22
Current	13	14	14	15
Body mass index, kg m^−2^	29.6 (3.9)	30.0 (3.9)	30.0 (3.8)	30.0 (3.8)
Waist-to-height ratio	0.6 (0.1)	0.6 (0.1)	0.6 (0.1)	0.6 (0.1)
Physical activity, METS-min day^−1^	270 (279)	237 (241)	224 (223)	194 (199)
Marital status, %				
Single	4	5	4	5
Married	81	75	77	72
Widowed	13	18	16	19
Other	2	3	4	3
Educational level, %				
Primary education or less	74	78	79	80
Secondary education	17	15	15	14
College or higher	9	7	6	6
Total energy intake, kcal day^−1^	2542 (535)	2347 (512)	2146 (460)	1909 (450)
Alcohol intake, g day^−1^	10 (15)	8 (14)	8 (14)	7 (13)
Protein intake, % energy	16.7 (2.7)	16.5 (2.7)	16.7 (2.8)	16.7 (3.0)
Carbohydrate intake, % energy	42.5 (7.1)	42.4 (6.9)	41.8 (7.1)	40.5 (7.3)
Total fat intake, % energy	38 (7)	39 (7)	39 (7)	40 (7)
Saturated, % energy	9 (2)	10 (2)	10 (2)	11 (2)
Monounsaturated, % energy	18 (5)	19 (4)	20 (4)	21 (5)
Polyunsaturated, % energy	7 (2)	6 (2)	6 (2)	6 (2)
Fiber, g day^−1^	34 (9)	27 (6)	23 (5)	17 (4)
Adherence to the Mediterranean diet (0 to 14 points)	10 (2)	9 (2)	8 (2)	8 (2)

All values are means (standard deviations), unless otherwise stated. When we adjusted for sex and age we did observe significant differences (*p* < 0.001) in age- and sex-adjusted average body mass index (BMI) across categories of the Dietary Inflammatory Index (DII). Participants in the highest category of the DII exhibited a significantly higher average BMI (30.23, 95% CI: 30.05–30.41) than those in the lowest quartile (BMI = 29.65, 95% confidence interval (CI): 29.47–29.83) after accounting for differences in sex and age. A similar direct and significant age-, and sex-adjusted association was observed for the waist-to-height ratio.

Adherence to the Mediterranean diet was inversely associated with the DII, being higher in the lowest DII quartile. However, the magnitude of the observed difference in Mediterranean diet adherence between extreme DII quartiles was not large.

Table 2 shows the hazard ratios (95% CI) for the risk of CVD, according to quartiles of the DII stratified by center and intervention group. When we adjusted for sex and age, the linear trend tests were statistically significant. Linear trend tests remained statistically significant (*p* < 0.05) after further adjustments for additional potential confounders. When we excluded events occurring within the first year of follow-up, we observed an even a stronger association with multivariable-adjusted HR above 1.90 for the two upper quartiles.

When we assessed the association between the DII and the incidence of CVD using the DII as a continuous variable (measured in standard deviation units), we observed that for each additional increase of 1 standard deviation in the DII, there was a 22% relative increase in the risk of CVD (95% CI: 6% to 40%) after multivariable adjustment.

Results were similar when we repeated the statistical analyses using quintiles instead of quartiles of adherence to the DII as the relevant exposure (data not shown, but available on request). There was no statistically significant interaction between the intervention arms (Mediterranean diet supplemented with either extra-virgin olive oil or Mediterranean diet supplemented with mixed nuts) and the DII. This indicates that the association between a more pro-inflammatory diet and a higher risk of CVD was fairly homogeneous across the three arms of the trial.

**Figure 2:**
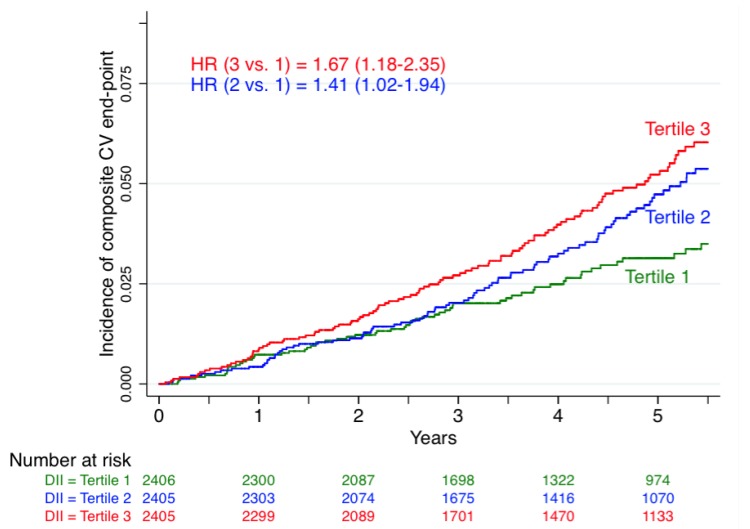
Incidence of cardiovascular disease according to tertiles of the dietary inflammatory index, the PREDIMED trial, 2003–2010.

Figure 2 shows the incidence of cardiovascular disease according to tertiles of the DII. Participants in the highest tertile of the DII (the most pro-inflammatory diet) had a significantly higher incidence of the composite cardiovascular end-point when compared with the lowest tertile.

**Figure 3:**
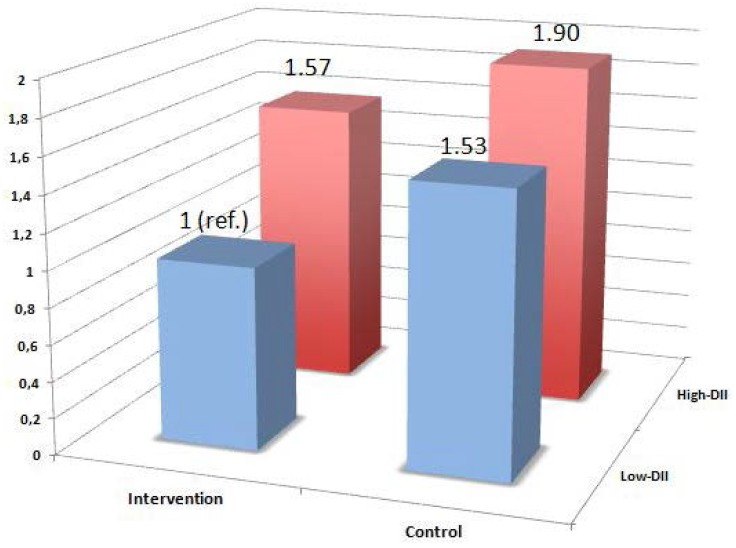
Cross-classification according to the PREDIMED intervention (both Mediterranean diet groups merged together) and to levels of the Dietary Inflammatory Index (DII) dichotomized by the median of the DII. Multivariable-adjusted Hazard Ratios for the primary end-point (a composite of myocardial infarction, stroke or cardiovascular death).

Figure 3 shows the cross-classification according to both intervention groups and the control group and to levels of DII dichotomized by the median of DII. The lowest risk of CVD was found in participants allocated to an active intervention with the Mediterranean diet and with low baseline values of the DII (representing a baseline anti-inflammatory dietary pattern), whereas the highest risk was observed in participants with a higher pro-inflammatory diet and allocated to the control group. Intermediate values of risk were found for participants in the control group with anti-inflammatory diets at baseline and in participants in the active intervention group with pro-inflammatory diets at baseline.

## 4. Discussion

This study provided evidence of a direct prospective association between increased diet-associated inflammation, indicated by a higher DII, and a higher risk of cardiovascular disease. The relationship showed a strongly linear, dose-response trend. This is consistent with the results reported by Ruiz-Canela *et al.*, in this same cohort showing that a higher DII was associated with higher levels of general obesity and abdominal obesity, after controlling for the effect that adherence to a MeDiet had on inflammation [12]. Three previous reports have shown a positive relationship between a higher DII and inflammation [18,19,20].

**Table 2:** Hazard ratios (95% confidence interval) for the risk of cardiovascular disease, according to the dietary inflammatory index, the PREDIMED trial, 2003–2010.

Hazard Ratios (HR)	Quartiles of Adherence to the Dietary Inflammatory Index				
	Q1	Q2	Q3	Q4	*P* for trend
Cases/person-years	49/7641	64/7755	85/7684	79/7960	
Crude HR	1 (ref)	1.32 (0.91–1.92)	1.84 (1.29–2.63)	1.68 (1.16–2.43)	0.003
Adjusted for age and sex HR	1 (ref)	1.41 (0.96–2.06)	1.87 (1.29–2.69)	1.76 (1.21–2.57)	0.002
Multivariable adjusted HR ^1^	1 (ref)	1.42 (0.97–2.09)	1.85 (1.27–2.71)	1.73 (1.15–2.60)	0.008
After excluding cases with follow-up < 1 year	36/7633	58/7752	73/7678	63/7951	
Crude HR	1 (ref)	1.62 (1.071–2.48)	2.16 (1.43–3.24)	1.83 (1.20–2.78)	0.005
Adjusted for age and sex HR	1 (ref)	1.75 (1.14–2.68)	2.21 (1.46–3.35)	1.93 (1.26–2.97)	0.004
Multivariable adjusted HR ^1^	1 (ref.)	1.76 (1.14–2.70)	2.22 (1.45–3.41)	1.90 (1.20–3.01)	0.012

1: adjusted for age and sex, overweight/obesity, waist-to-height ratio, total energy intake (quartiles), smoking status (3 categories), diabetes, hypertension, dyslipidemia, family history of premature cardiovascular disease, physical activity (quartiles) and educational level, and stratified by intervention group and center. All models were stratified by intervention group and center.

An interesting finding in our study is that we observed a strengthening of the association when we excluded events occurring within the first year of follow-up. This is consistent with our initial hypothesis because very early events may be related to other previous exposures and not necessarily to the DII measured at baseline, *i.e.*, immediately before those events. As it would be unlikely to assume a very short induction period for the association between the DII and the occurrence of new CVD clinical events, our expectation was to find a stronger association when very early events were removed.

The association between DII scores, obtained from a food-frequency questionnaire (FFQ), and serum levels of inflammatory biomarkers measured in other cohorts have been reported [18,19,20,21]. However, we did not measure these biomarkers in all participants in our cohort and therefore we were not able to assess this association. One advantage of using the DII instead of biomarkers is that we could comment on the direct association between dietary exposures and clinical events. Additional advantages include reduced cost and the avoidance of blood collection and analytical determinations. By using a simple, inexpensive, noninvasive tool (the FFQ)we were able to assess the role of diet associated inflammation without relying on an intermediate measure. .

Diet has consistently been shown to regulate inflammation [22,23,24,25]. Specifically, a modified version of the DII has recently been shown to predict a higher summary score for inflammation according to a combination of six inflammatory serum biomarkers, and to predict markers of glucose metabolism [26]. However, a study from Luxembourg reported no association between the same new, improved DII that we used in this study and diastolic blood pressure, CRP, lipids, and glycemic biomarkers [27].

In previous analyses of the PREDIMED study, an increased adherence to the Mediterranean diet had been shown to be associated with lower levels of CRP. The PREDIMED study also has found lower levels of other inflammatory and immune biomarkers associated with better adherence to the Mediterranean diet, a higher consumption of some of the typical Mediterranean foods or with the interventions conducted in this trial [5,28,29,30,31]. The inverse association between the DII and adherence to the Mediterranean diet observed in our data is consistent with the predicted association between DII and CRP showed by Shivappa *et al.*, in the SEASONS and the Asklepos studies [18,19] and by Wirth *et al.*, among police officers [32]. Other studies have reported that higher adherence to the Mediterranean diet is associated with decreased levels of inflammatory biomarkers (including CRP, interleukin-6, and intracellular adhesion molecule-1) as summarized by a recent meta-analysis [33]. This consistency in findings provides further strength to our results.

Additional strengths of our study are that: (a) we used a prospective follow-up design to ascertain the occurrence of clinical events; (b) our analyses were not mainly based in intermediate biomarkers changes but provided direct evidence of an association with final hard clinical events; (c) we were able to control for a wide array of potentially confounding factors and to assess the combined effect of the baseline inflammatory potential of the diet and the dietary intervention.

Some limitations need also to be acknowledged. Not all foods of the FFQ were included in the calculation of the DII. The DII was built using articles that examined the effect of individual nutrients in relation to intermediate biomarkers, but it did not assess the effect of the overall dietary pattern intake on these biomarkers. As the DII was created depending on the published literature, there could be findings that were not included or not published because of null findings (*i.e.*, publication bias may have occurred). In any case, the DII was created based on an extensive literature search and did not take into account only certain nutrients or foods but tried to assess many of them, and, therefore, in this way did assess the whole diet. Also, there were a large number of null results reported in the reviewed literature. Other limitations are related to the generalizability of our findings. Because all study participants lived in a Mediterranean country and were at high cardiovascular risk, extrapolating our results requires replication in other settings and populations. In any case, the fact is that participants in the PREDIMED study, despite being high-risk subjects, had an average lower DII (−0.75, SD: 1.53) than participants in previous studies that have assessed the inflammatory capacity of the diet using the DII (+0.84 SD: 1.99) [19].

## 5. Conclusions

In summary, we found for the first time prospective direct evidence that diets with higher pro-inflammatory potential were directly associated with a increased likelihood of developing clinical cardiovascular events. This direct association was strong, gradual, and consistent across different methods of categorizing the dietary inflammatory capacity and showed a linear dose-response trend.

## Declaration

James R. Hébert owns controlling interest in Connecting Health Innovations LLC (CHI), a company planning to license the right to his invention of the dietary inflammatory index (DII) from the University of South Carolina in order to develop computer and smart phone applications for patient counseling and dietary intervention in clinical settings. Nitin Shivappa is an employee of CHI. The subject matter of this paper will have no direct bearing on the work of CHI, nor has any CHI-related activity exerted any influence on this project. Other authors declare no conflict of interest.

## References

[1] World Health Organization (2012). World Health Statistics.

[2] Ross R. (1999). Atherosclerosis—An inflammatory disease. N. Engl. J. Med..

[3] Páramo J., Rodríguez J., Orbe J. (2007). Atherosclerosis in inflammatory diseases. Med. Clin..

[4] Lopez-Garcia E., Schulze M.B., Fung T.T., Meigs J.B., Rifai N., Manson J.E., Hu F.B., Schulze M. (2004). Major dietary patterns are related to plasma concentrations of markers of inflammation and endothelial dysfunction. Am. J. Clin. Nutr..

[5] Salas-Salvadó J., Garcia-Arellano A., Estruch R., Marquez-Sandoval F., Corella D., Fiol M., Gómez-Gracia E., Viñoles E., Arós F., Herrera C. (2008). Components of the Mediterranean-type food pattern and serum inflammatory markers among patients at high risk for cardiovascular disease. Eur. J. Clin. Nutr..

[6] Esmaillzadeh A., Kimiagar M., Mehrabi Y., Azadbakht L., Hu F.B., Willett W.C. (2007). Dietary patterns and markers of systemic inflammation among Iranian women. J. Nutr..

[7] Fung T., Rexrode K.M., Mantzoros C.S., Manson J.E., Willett W.C., Hu F.B. (2009). Mediterranean diet and incidence of and mortality from coronary heart disease and stroke in women. Circulation.

[8] Martínez-González M., García-López M., Bes-Rastrollo M., Toledo E., Martínez-Lapiscina E.H., Delgado-Rodriguez M., Vazquez Z., Benito S., Beunza J.J. (2011). Mediterranean diet and the incidence of cardiovascular disease: A Spanish cohort. Nutr. Metab. Cardiovasc. Dis..

[9] Trichopoulou A., Martínez-González M.A., Tong T.Y., Forouhi N.G., Khandelwal S., Prabhakaran D., Mozaffarian D., de Lorgeril M. (2014). Definitions and potential health benefits of the Mediterranean diet: Views from experts around the world. BMC Med..

[10] Martinez-Gonzalez M.A., Bes-Rastrollo M. (2014). Dietary patterns, Mediterranean diet, and cardiovascular disease. Curr. Opin. Lipidol..

[11] Shivappa N., Steck S.E., Hurley T.G., Hussey J.R., Hébert J.R. (2014). Designing and developing a literature-derived, population-based dietary inflammatory index. Public Health Nutr..

[12] Ruiz-Canela M., Zazpe I., Shivappa N., Hébert J.R., Sánchez-Tainta A., Corella D., Salas-Salvadó J., Fitó M., Lamuela-Raventós R.M., Fernández-Crehuet J. (2015). Dietary inflammatory index and anthropometric measures of obesity in a population sample at high cardiovascular risk from the PREDIMED trial. Br. J. Nutr..

[13] Martínez-González M., Corella D., Salas-Salvadó J., Ros E., Covas M.I., Fiol M., Wärnberg J., Arós F., Ruíz-Gutiérrez V., Lamuela-Raventós R.M. (2012). Cohort profile: Design and methods of the PREDIMED study. Int. J. Epidemiol..

[14] Ros E., Martínez-Gonz;lez M.A., Estruch R., Salas-Salvadó J., Fitó M., Martínez J.A., Corella D. (2014). Mediterranean diet and cardiovascular health: Teachings of the PREDIMED study. Adv. Nutr..

[15] Estruch R., Ros E., Salas-Salvadó J., Covas M.I., Corella D., Arós F., Gómez-Gracia E., Ruiz-Gutiérrez V., Fiol M., Lapetra J. (2013). Primary prevention of cardiovascular disease with a Mediterranean diet. N. Engl. J. Med..

[16] Zazpe I., Sanchez-Tainta A., Estruch R., Lamuela-Raventos R.M., Schröder H., Salas-Salvado J., Corella D., Fiol M., Gomez-Gracia E., Aros F. (2008). A large randomized individual and group intervention conducted by registered dietitians increased adherence to Mediterranean-type diets: The Predimed study. J. Am. Diet. Assoc..

[17] Tabung F., Steck S.E., Ma Y., Liese A.D., Zhang J., Caan B., Hou L., Johnson K.C., Mossavar-Rahmani Y., Shivappa N. (2015). The association between dietary inflammatory index and risk of colorectal cancer among postmenopausal women: Results from the Women’s Health Initiative. Cancer Causes Control.

[18] Shivappa N., Hébert J.R., Rietzschel E.R., de Buyzere M.L., Langlois M., Debruyne E., Marcos A., Huybrechts I. (2015). Associations between dietary inflammatory index and inflammatory markers in the Asklepios Study. Br. J. Nutr..

[19] Shivappa N., Steck S.E., Hurley T.G., Hussey J.R., Ma Y., Ockene I.S., Tabung F., Hébert J.R. (2014). A population-based dietary inflammatory index predicts levels of C-reactive protein in the Seasonal Variation of Blood Cholesterol Study (SEASONS). Public Health Nutr..

[20] Wood L.G., Shivappa N., Berthon B.S., Gibson P.G., Hebert J.R. (2015). Dietary inflammatory index is related to asthma risk, lung function and systemic inflammation in asthma. Clin. Exp. Allergy.

[21] Tabung F.K., Steck S.E., Zhang J., Ma Y., Liese A.D., Agalliu I., Hingle M., Hou L., Hurley T.G., Jiao L. (2015). Construct validation of the dietary inflammatory index among postmenopausal women. Ann. Epidemiol..

[22] Khan N., Khymenets O., Urpí-Sardà M., Tulipani S., Garcia-Aloy M., Monagas M., Mora-Cubillos X., Llorach R., Andres-Lacueva C. (2014). Cocoa polyphenols and inflammatory markers of cardiovascular disease. Nutrients.

[23] Wang Y., Chun O.K., Song W.O. (2013). Plasma and dietary antioxidant status as cardiovascular disease risk factors: A review of human studies. Nutrients.

[24] Gariballa S., Kosanovic M., Yasin J., El Essa A. (2014). Oxidative damage and inflammation in obese diabetic Emirati subjects. Nutrients.

[25] Hermsdorff H.H., Zulet M.Á., Abete I., Martínez J.A. (2011). A legume-based hypocaloric diet reduces proinflammatory status and improves metabolic features in overweight/obese subjects. Eur. J. Nutr..

[26] Van Woudenbergh G., Theofylaktopoulou D., Kuijsten A., Ferreira I., van Greevenbroek M.M., van der Kallen C.J., Schalkwijk C.G., Stehouwer C.D., Ocké M.C., Nijpels G. (2013). Adapted dietary inflammatory index and its association with a summary score for low-grade inflammation and markers of glucose metabolism: The Cohort study on Diabetes and Atherosclerosis Maastricht (CODAM) and the Hoorn study. Am. J. Clin. Nutr..

[27] Alkerwi A., Shivappa N., Crichton G., Hébert J.R. (2014). No significant independent relationships with cardiometabolic biomarkers were detected in the Observation of Cardiovascular Risk Factors in Luxembourg study population. Nutr. Res..

[28] Casas R., Sacanella E., Urpí-Sardà M., Chiva-Blanch G., Ros E., Martínez-González M.A., Covas M.I., Lamuela-Raventos R.M., Salas-Salvadó J., Fiol M (2014). The effects of the mediterranean diet on biomarkers of vascular wall inflammation and plaque vulnerability in subjects with high risk for cardiovascular disease. A randomized trial. PLoS ONE.

[29] Bulló M., Casas R., Portillo M.P., Basora J., Estruch R., García-Arellano A., Lasa A., Juanola-Falgarona M., Arós F., Salas-Salvadó J. (2013). Dietary glycemic index/load and peripheral adipokines and inflammatory markers in elderly subjects at high cardiovascular risk. Nutr. Metab. Cardiovasc. Dis..

[30] Estruch R., Martínez-González M.A., Corella D., Salas-Salvadó J., Ruiz-Gutiérrez V., Covas M.I., Fiol M., Gómez-Gracia E., López-Sabater M.C., Vinyoles E. (2006). Effects of a Mediterranean-style diet on cardiovascular risk factors: A randomized trial. Ann. Intern. Med..

[31] Mena M., Sacanella E., Vazquez-Agell M., Morales M., Fitó M., Escoda R., Serrano-Martínez M., Salas-Salvadó J., Benages N., Casas R. (2009). Inhibition of circulating immune cell activation: A molecular antiinflammatory effect of the Mediterranean diet. Am. J. Clin. Nutr..

[32] Wirth M., Burch J., Shivappa N., Violanti J.M., Burchfiel C.M., Fekedulegn D., Andrew M.E., Hartley T.A., Miller D.B., Mnatsakanova A. (2014). Association of a dietary inflammatory index with inflammatory indices and metabolic syndrome among police officers. J. Occup. Environ. Med..

[33] Schwingshackl L., Hoffmann G. (2014). Mediterranean dietary pattern, inflammation and endothelial function: A systematic review and meta-analysis of intervention trials. Nutr. Metab. Cardiovasc. Dis..

